# Preparation of a PANI/ZnO Composite for Efficient Photocatalytic Degradation of Acid Blue

**DOI:** 10.3390/polym10090940

**Published:** 2018-08-23

**Authors:** Vanja Gilja, Ivan Vrban, Vilko Mandić, Mark Žic, Zlata Hrnjak-Murgić

**Affiliations:** 1 Faculty of Chemical Engineering and Technology, University of Zagreb, Marulićev trg 19, 10000 Zagreb, Croatia; vgilja@fkit.hr (V.G.); ivrban@fkit.hr (I.V.); vmandic@fkit.hr (V.M.); 2 Division of Materials Physics, Ruđer Bošković Institute, Bijenička cesta 54, 10000 Zagreb, Croatia

**Keywords:** in situ synthesis, PANI/ZnO composite, conductive polymer, photocatalyst, solar photocatalysis, acid blue dye

## Abstract

Polyaniline/zinc oxide (PANI/ZnO) composite photocatalysts were prepared from neutral media by in situ chemical oxidation of aniline (ANI) in the presence of different amounts of diethylene glycol (DEG). The PANI/ZnO composite photocatalysts were synthesized to efficiently remove organic dye (acid blue, AB25) from model wastewater. The PANI/ZnO composite photocatalysts were studied with the intention of efficient removal of organic dye (acid blue, AB25) from wastewater to obtain low-cost heterogeneous catalysts that offer high catalytic activity and stability. The conductive PANI polymer, which absorbs Vis irradiation, was used in this work as ZnO absorbs only ultraviolet (UV) irradiation; thus, the composite photocatalysts’ activity was broadened into the Vis region. Characterization of the composite photocatalysts was done by Fourier transform infrared spectroscopy, X-ray diffraction, thermogravimetric analysis, scanning electron microscopy, electric conductivity, UV-Vis spectroscopy, and by specific surface area (SBET) measurements. The composites’ photocatalytic activity under solar irradiation was validated by monitoring degradation of the AB25 dye. This study presented that it was possible both to prepare PANI and to prevent ZnO dissolution if in situ polymerization starts from neutral media with the addition of DEG. Additionally, efficient removal of AB25 dye, about 90% in 60 min, was achieved. The first-order rate constants of the photodegradation of AB25 by PANI/ZnO 0.02/0.024/0.04 DEG (and pure ZnO)) were computed to be 0.0272/0.0281/0.0325 (and 0.0062) min^−1^, indicating that the morphology and surface of the photocatalysts have significantly influenced the catalytic activity.

## 1. Introduction

Increased awareness regarding a material’s performance and increased competition in the global market for polymer composites have fueled their growth. Composites (i.e., the wonder materials) are becoming an essential part of today’s materials due to the construction of more complex hybrid materials. Nanoarchitectonics is an emerging field that refers to the rational design and construction of nanostructures into functional materials with control at the nanoscale [1,2,3].

It is now recognized that materials are often the limiting factor in bringing a new technical concept to fruition; thus, polymers are often materials of choice in these demanding applications. Furthermore, composites can be defined as materials consisting of two or more chemically and physically different phases that are separated by a distinct interface. Therefore, different materials are combined judiciously to achieve a system (i.e., composite) with more useful structural or functional properties that are unattainable by any of the constituents alone.

To obtain superior composites properties the interfacial adhesion should be strong. This can be achieved on the molecular level by a chemical reaction or adsorption [4]. In situ preparation of polymer composites is one very successful method to achieve high interfacial interactions between two materials. Polyaniline/zinc oxide (PANI/ZnO) composite photocatalysts are usually prepared to overcome some of limiting factors of zinc oxide, which is usually used as an alternative to a titanium oxide (TiO_2_) photocatalyst.

The main barriers for extensive ZnO applicability are the narrow light-absorption range, the charge-carrier recombination, the photoinduced corrosion–dissolution at extreme pH conditions, and the formation of inert Zn(OH)_2_ during photocatalytic reactions. To overcome the aforementioned obstacles and to enhance photocatalysis under ambient conditions, several authors modified the surface and structure of ZnO [5,6,7,8,9]. Hence, a variety of efforts have been made to solve ZnO-related problems by, e.g., morphology control [10], metal or metal-oxide doping [11,12], and hybrid composites with other materials [13,14].

PANI was chosen as one of the promising conductive polymers to tune composites’ optical, electrical, and photocatalyst properties [15,16]. The advantages of preparing PANI/ZnO composite photocatalysts in neutral media with diethylene glycol (DEG) additions are: (i) improved aniline solubility in neutral aqueous solution and more stable dispersion, (ii) the ability to avoid dissolution of ZnO during in situ synthesis, (iii) the ability to enable photocatalysis under solar irradiation due to a diminished bandgap, and (iv) the ability to prevent corrosion-dissolution of ZnO during photocatalysis.

However, the challenge is to find experimental conditions that result in encapsulation of ZnO nanoparticles [17], which can be achieved by allowing for the preparation of conductive PANI and by preventing ZnO dissolution. Furthermore, polymerization from neutral media brings a concern about PANI charge transfer properties because highly oxidative polymer chains can be obtained only in acid media [18]. Please remember that in the fully reduced state the polymer is an insulator, whereas it is the most stable and conductive in the half-oxidized state [19,20].

Furthermore, the charge transfer is governed by radical cations (polaron) or dications (bipolaron) that are delocalized over several units of the polymers [21,22]. The higher radical density leads to the greater electrical conductivity that governs photocatalysts’ properties. PANI has an ability to inject e_CB_^−^ (obtained by absorption of a photon from Vis irradiation) into the ZnO conduction band (CB), and at the same time it can accept h_VB_^+^ from the ZnO valence band (VB). Thus, the obtained iterations yield a PANI-ZnO synergistic effect, which hinders the recombination process in ZnO and increases activity under Vis radiation [23].

Afterward, the typical photocatalysis mechanism for metal-oxides proceeds, i.e., e_CB_^−^ and h_VB_^+^ transform H_2_O and O_2_ into OH^−1^ radicals which in turn degrade the organic pollutants [24,25]. Furthermore, many organic pollutants (e.g., dyes) are industrial effluents generated from various sources [26,27,28]. So, photocatalysis has emerged as a potential strategy to degrade dyes in wastewater [29,30]. To improve photocatalytic properties and to extend the pure TiO_2_ (3.2 eV) absorption to the visible light region, several authors have prepared carbon-doped TiO_2_, which has a lower bandgap (≈3.01 eV) [31,32,33]. However, the recombination process within TiO_2_ was not solved. As an alternative to TiO_2_, ZnO has also been studied as a potential candidate for wastewater treatment due to, e.g., its similarity in charge carrier dynamics upon bandgap excitation.

The purpose of this work is to prevent the dissolution of ZnO particles during the in situ chemical oxidation of aniline and to prepare a PANI/ZnO composite photocatalyst. Consequently, the photocatalytic activity of the pure ZnO is extended to the region of solar radiation. The properties of the photocatalyst were studied in detail and the photodegradation performance of PANI/ZnO catalysts was validated by degradation of acid blue dye (AB25) in water.

## 2. Materials and Methods

### 2.1. Materials

Zinc oxide (ZnO), nanopowder, particle size <100 nm, Sigma-Aldrich (Saint Louis, MO, USA); aniline (An, 99%, Morris Plains, NJ, USA) and ammonium persulfate (APS, (NH_4_)_2_S_2_O_8_, Morris Plains, NJ, USA) Acros Organics; and sulfuric acid (H_2_SO_4_, 96%, Kemika, Zagreb, Crioatia) and diethylene glycol (DEG, C_4_H_10_O_3_, Kemika, Zagreb, Crioatia) were used to synthesize composite samples. Commercial organic dye C.I. Acid Blue 25 (AB25) Ciba-Geigy was used as the water pollutant. The chemicals were used as received, without any additional pre-treatment. All aqueous solutions were prepared with ultrapure water from the Merc Millipore Direct-Q3 UV water purification system (Merc KGaK, Darmstadt, Germany).

One of ZnO’s main drawbacks is a photocorrosion reaction under ultraviolet A (UVA) light, which can be presented by Equation (1) [34,35]:

(1) $$\mathrm{ZnO}+2h^{+}+nH_{2}O\;\to \mathrm{Zn}{\left(\mathrm{OH}\right)}_{n}^{{\left(2-n\right)}^{+}}+1/2O_{2}+nH^{+}$$

where *n* depends on the pH of the media and *h^+^* is photo-induced hole (see, e.g., Section 3.8).

However, intense ZnO solubility in aqueous solutions also prevents its wider application.

In strong acid media:

(2) $$\;\mathrm{ZnO}+2H^{+}\to {\mathrm{Zn}}^{2+}+1/2O_{2}$$

(3) $$\;\mathrm{ZnO}+2H^{+}\to {\mathrm{Zn}}^{2+}+H_{2}O$$

In an alkaline medium:

(4) $$\;\mathrm{ZnO}+H_{2}O+2{\mathrm{OH}}^{-}\to \mathrm{Zn}{\left(\mathrm{OH}\right)}_{4}^{2-}$$

Upon UV irradiation:

(5) $$\;2\mathrm{ZnO}+4H_{2}O+4h^{+}\to 2\mathrm{Zn}{\left(\mathrm{OH}\right)}_{2}+O_{2}+4H^{+}$$

Thus, both strongly acidic and alkaline conditions do not favor both the composite’s synthesis and photocatalytic process.

### 2.2. Composite Preparation

Polyaniline/zinc oxide (PANI/ZnO) composites preparation was conducted by in situ chemical oxidation polymerization at room temperature whilst the starting pH media of a water and diethylene glycol (DEG) mixture was neutral. The aniline monomer was oxidized by APS in the presence of ZnO nanoparticles. The constant mole ratio of monomer and oxidant was n(An):n(APS) = 1:0.25, the weight ratio of m(PANI):m(ZnO) was 15:85 for all prepared samples, and the concentration of DEG was 0.02, 0.024, and 0.04 M. Samples are denoted as: PANI/ZnO 0.02 DEG, PANI/ZnO 0.024 DEG, and PANI/ZnO 0.04 DEG. The procedure for the PANI/ZnO 0.02 DEG sample was as follows: solution A was prepared using 0.8 g ZnO nanoparticles, 0.386 mL of DEG, and 0.588 mL of aniline in 25 mL distilled water and sonicated for 15 min to obtain a stable suspension. Solution B consisted of 0.367 g APS in 25 mL distilled water (Table 1). An An-ZnO suspension (solution A) was mixed and stirred (250 rpm) in a reactor container for an additional period of 15 min, and after that, the solution B was added in reactor container and in situ polymerization was started and carried out for 2 h. The prepared composite was washed (H_2_O) several times, separated by the centrifuge, and dried at 60 °C for 24 h.

### 2.3. Characterizations

Fourier transform infrared (FTIR) spectroscopic data were obtained with a Perkin-Elmer Spectrum One FT-IR spectrometer (Perkin Elmer, Shelton, CT, USA) equipped with an attenuated total reflection accessory (ATR with ZnSe diamond crystal) in reflection mode. The spectra were collected in the range 650–4000 cm^−1^ with a resolution of 4 cm^−1^.

UV-Vis spectra of powdery samples of PPy/ZnO composites were recorded by a Shimadzu UV-Vis-near infrared (NIR) (UV-3600) spectrometer (Shimadzu Corporation, Tokyo, Japan) with the integrated sphere. Barium sulfate was used as the reference material.

The morphology of the studied samples was analyzed using scanning electron microscope (SEM) images obtained by a thermal field emission scanning microscope (JEOL Ltd., Akishima, Japan) (FE SEM, model JSM-7000F) operating at 3 kV.

Thermal properties of the composites were analyzed by thermogravimetric analysis (TGA) using a TA Instruments Q500 analyzer (TA Instruments, New Castle, DE, USA). TGA curves were obtained under a nitrogen atmosphere (40 mL/min) and a heating rate of 10 °C/min from room temperature to 800 °C. The derivative thermogravimetric (DTG) curves were directly derived from the original TGA thermogram using the product software. 

Conductivity of PANI/ZnO composites was determined by measuring their electrical resistance with the four-point probe method (Keysigiht 34461 61/2 Digit Multimeter, Santa Rosa, CA, USA). Electrical resistance is calculated according to Equation (6):

*ρ* = (2*πdR*)/ln2 (6)

where *ρ* is the specific electrical resistance (Ω cm), *R* is the electrical resistance (Ω), and *d* is the thickness of pastilles (cm). The electric conductivity (κ/S cm^−1^) was calculated as a reciprocal value of the electrical resistance (Equation (7)):

*κ* = 1/*ρ*. (7)

**Scheme 1:**
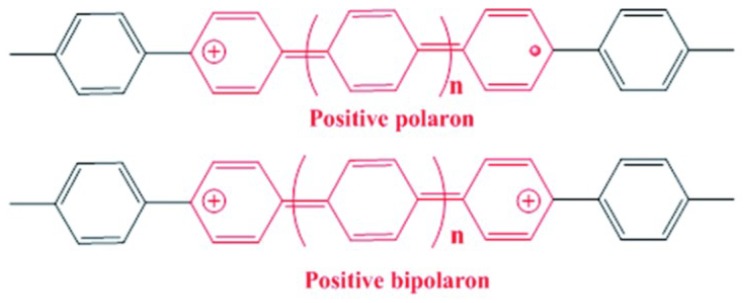
Polyaniline (PANI) polymer in conductive (i.e., half-oxidized) state. The presented polaron and bipolaron govern PANI properties.

For each sample, at least 10 measurements were made and results are given as their average value [36]. Note that PANI properties (e.g., conductivity) are governed by radical cations (polaron) or dications (bipolaron) (Scheme 1).

Prepared samples were characterized by powder X-ray diffraction (PXRD) at room temperature (RT) using a Shimadzu XRD6000 diffractometer device (Shimadzu Corporation, Tokyo, Japan with monochromatized Cu K radiation under a voltage acceleration of 40 kV and a current of 30 mA. For each prepared sample, the data were collected in a step scan of 2–70° 2*θ* with steps of 0.02° 2*θ* and a counting time of 0.6 s. The fits were carried out by means of a split-type pseudo-Voigt profile function and the polynomial background model. The crystallite size was determined using Scherrer’s expression. Isotropic vibration modes were assumed for all atoms. The instrumental line broadening was determined using LaB_6_ powder.

Nitrogen (N_2_) adsorption isotherms were obtained at −196 °C using Gemini 2380 (Micromeritics Instrument Corporation, Norcross, GA, USA). The Brunauer-Emmett-Teller (BET) specific surface area (*S*_BET_) of composites was calculated using N_2_ adsorption isotherms. All the samples were dried at 50 °C for 1 h and degassed under vacuum at 400 °C for 24 h prior to analysis.

### 2.4. Photodegradation of Acid Blue Dye

The photocatalytic properties of the PANI/ZnO composite were determined by monitoring the photodegradation of AB25 dye in aqueous solution after exposing it to an Oriel Newport solar irradiation simulator (Osram XBO 450 W lamp) (Oriel Instruments, Irvine, CA, USA). In a typical procedure, 50 mg/L of the PANI/ZnO composite was added into 50 mL aqueous solution of AB25 (30 mg/L), and the pH media was adjusted to 7. Then, the mixture was kept in the dark with constant shaking for 30 min to reach the adsorption-desorption equilibrium. Furthermore, the irradiation was carried out at a horizontal lamp-sample distance of 25 cm and the photocatalytic process was carried out for 60 min. At given intervals (15 min), an aliquot of photoreacted AB25 solutions was taken and was filtered (Chromafil XTRA RC (25 mm, 0.45 micron) filters (Macherey-Nagel, Düren, Germany)) and then subjected to UV-Vis spectroscopic measurements to determine the AB25 concentration. The degradation of AB25 was determined by the intensity of the absorption peak at 622 nm using a Perkin Elmer Lambda EZ 201 spectrophotometer (Perkin Elmer, Shelton, CT, USA). The results were presented as the concentration of remaining dye in the wastewater after treatment according to the equation:

(8) $$Color=\frac{\left(A_{0}-A_{t}\;\right)}{A_{0}}\cdot 100\%$$

where *A*_0_ and *A_t_* are the adsorption values at *t* = 0 and *t* = *t*, respectively.

## 3. Results and Discussion

### 3.1. FTIR Characterization

**Figure 1:**
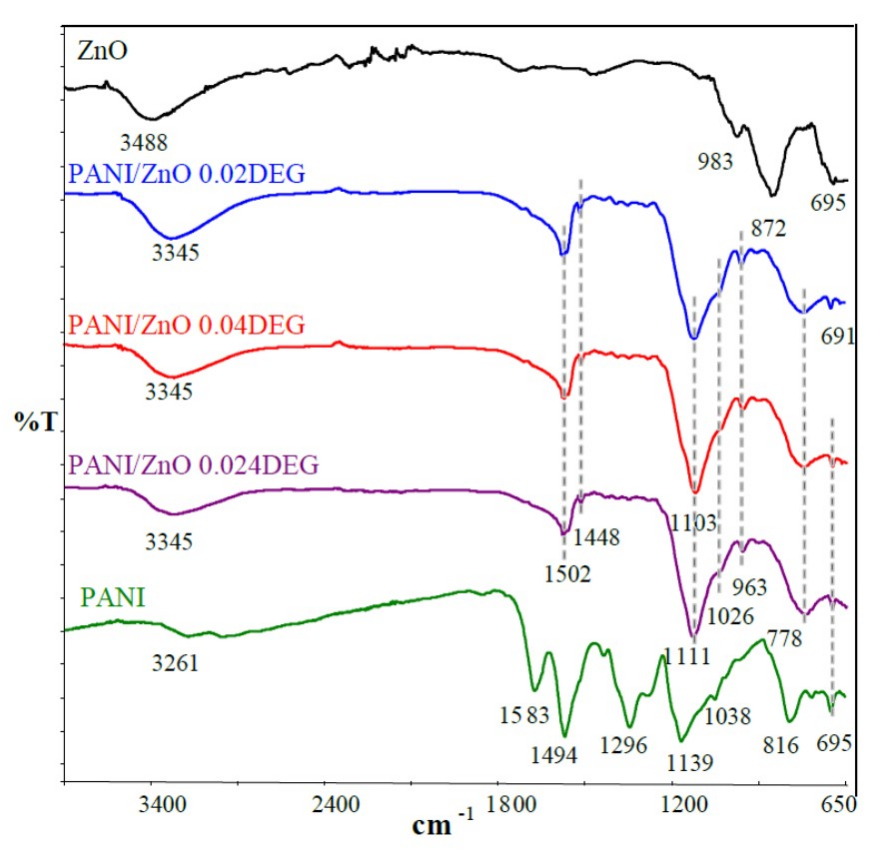
Fourier transform infrared (FTIR) spectra of pure polyaniline (PANI), zinc oxide (ZnO), and PANI/ZnO composites.

**Scheme 2:**
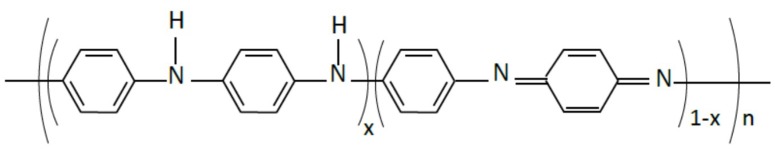
The structural formula of PANI benzoid (B) and quinoid (Q) units, i.e., [(–B–NH–B–NH–)*_x_* –(–B–N=Q=N–)_(1−*x*)_]*_n_*.

FTIR spectra of pure PANI, ZnO and PANI/ZnO composites are given in Figure 1, in which the most prominent absorption bands correspond to the PANI polymer. The main absorption peaks are positioned at 3261, 1583, 1494, and 1296 cm^−1^ and they can be attributed to N–H, C=N, C=C, and C–N stretching, respectively. The absorption intensity ratio of PANI bands (1583 and 1494 cm^−1^) typical for quinoid (Q) and benzoid (B) units is 1:3. This ratio is in accordance to the Q versus B units ratio (1:3) in Scheme 2 [37]), which indicates that PANI (Figure 1) is in a conductive form. Note that the oxidation of aniline by APS [20] also results in an excess of protons that enabled the formation of Q units.

Furthermore, the pure PANI shows bands at 1139 and 816 cm^−1^, which correspond to C–H stretching [38]. In the case of PANI/ZnO composites, the N–H, C=N, C=C, and C–N stretching bands were slightly shifted and they are observed at 3345, 1502, 1448, and 1103 cm^−1^, respectively. Additionally, diethylene glycol (DEG) induced bands at 1026 and 963 cm^−1^ (i.e., C–O–C stretching) and at 778 cm^−1^ (–CH_2_), which confirms its presence in composites even after washing.

The FTIR spectrum of ZnO shows an intensive band at 3488 cm^−1^, which represents the stretching vibrations of water molecules [39]. The absorption bands in the low frequency region of spectra located in the range 431–872 cm^−1^ characterize Zn–O–Zn and Zn–O stretching. The shift of characteristic ZnO bands in PANI/ZnO composites is due to interactions between PANI and ZnO nanoparticles. Also, a disappearance of the absorption band at 872 cm^−1^, which is typical for ZnO composites, suggests that PANI encapsulated ZnO and/or it was immobilized on the ZnO surface.

### 3.2. Conductivity of PANI/ZnO Composites

The conductivity of PANI/ZnO composites synthesized with a different concentration of DEG was determined by the four-point method. The conductivity (κ/S cm^−1^) was computed by using Equations (6) and (7) and the corresponding values are given in Table 2. It is apparent that ZnO has a significantly lower value (2.955 × 10^−7^ S cm^−1^) than the composite samples (≈10^−6^ S/cm^−1^). Increased conductivity confirms the presence of a relatively well-conducting PANI polymer, which was prepared from a neutral aqueous medium with the addition of DEG. This result agrees with the literature as it is known that PANI synthesized in neutral media has poor conductivity [40], while its conductivity considerably increases during the synthesis in acid media (see, e.g., [41]).

Furthermore, it is expected that increased PANI conductivity in composites will enhance interactions between PANI and ZnO. The PANI conductivity in composites shows slight variations due to the same composition (see thermogravimetric (TG) results in Table 3). The composite samples differ only in the fraction of DEG, which perturbed the structure of polymer chains. Thus, the lowest conductivity of PANI/ZnO 0.040 DEG may indicate that the main polymer chains are of a low degree of order [42,43] or its surface is more covered by ZnO nanoparticles as presented in the SEM micrographs.

### 3.3. Thermogravimetric Analysis Characterization

**Figure 2:**
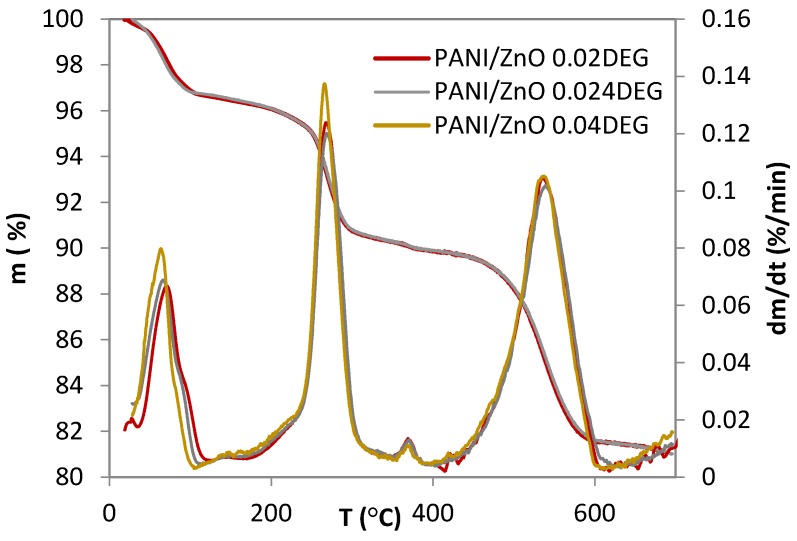
Thermogravimetric (TG) and derivative thermogravimetric (DTG) curves of PANI/ZnO composites.

TGA was conducted to determine the thermal stability and the composition of the prepared PANI/ZnO composites. The PANI/ZnO weight loss was monitored in the range of 40–800 °C and the results are given in Figure 2 and Table 3. TG curves profiles showed three distinct temperature regions (i.e., weight loss). The first temperature region, which started from ≈60 °C, occurred due to DEG evaporation and due to the loss of physically adsorbed water. The second weight loss that begins at ≈230 °C (and the minor maximum at 365 °C) can be attributed to the removal of impurities (i.e., removal of oligomers and amino groups in the *ortho*-position) [44]. The third temperature region (>400 °C) was governed by the degradation of the PANI chains in a nitrogen atmosphere, which confirms the presence of the conductive PANI [45].

Table 3 shows the weight losses for each specific region and they are approximately the same for each composite. As one can observe, the PANI mass in the composites is ≈8.4% and the char residue is ≈77% for all three samples. This indicates that the same composition was obtained, which denotes high reproducibility of the PANI synthesis in neutral to slightly acidic media in spite of the variation in DEG concentration. The temperatures at the maximum degradation rate (*T_max_*_2,3,4_) obtained from the DTG profiles (see peaks at ~268, ~365, and ~530 °C) show some variation (see Figure 2 and Table 3). The DTG data suggest that a difference in PANI chain structure induced the slight variation in thermal stability [25]. Also, it can be concluded that the presence of DEG hindered dissolution of ZnO during the in situ aniline oxidation by APS, which slowly decreases the pH of polymerization media.

### 3.4. Morphology of PANI/ZnO Composites

**Figure 3:**
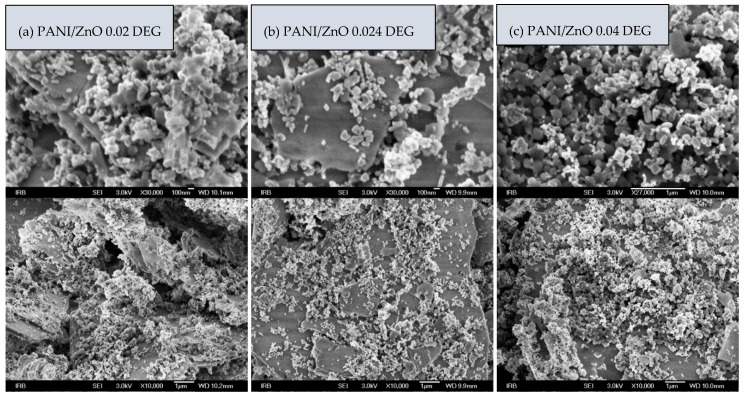
FE SEM images of the PANI/ZnO composites at different magnifications (**up**) X30.000 and (**down**) X10.000. Images show the impact of different diethylene glycol (DEG) concentrations on in situ composites synthesis.

Figure 3 shows FE SEM images of the PANI/ZnO composite at different magnifications. The images with lower magnification clearly show the dual structure of the PANI/ZnO composite that is comprised of spherical-granular particles and lamellas. The composites consist of ZnO granules (≈100–200 nm in diameter) which are bound to the surface of lamellar structures [46]. The images with the higher magnification clearly present the effect of different DEG concentrations, i.e., an increase in DEG concentration yielded a higher number of the spherical particles, whilst the number of lamellas decreased.

The highest fraction of spherical particles was observed for the PANI/ZnO 0.04 DEG sample. The particles were homogeneously dispersed on the lamellar structure indicating clear PANI/ZnO morphological transformations from lamellas to spherical-shaped granules. However, the lamellas to granules transformation should increase the catalysts’ specific surface as well as the number of the synergistic spots (i.e., interactions) in PANI/ZnO. Therefore, this DEG-induced transformation should additionally boost photocatalysts’ properties, i.e., it should increase the efficiency of PANI/ZnO composites.

### 3.5. Surface Area Analysis

The specific surface area (BET) values were computed using nitrogen adsorption isotherms of PANI/ZnO composites and they are presented in Table 4. An increase in the specific surface area was observed when the samples were synthesized with the higher DEG concentration. This correlates well with the FE SEM results, which indicate that PANI/ZnO 0.04 DEG contains a significantly higher fraction of spherical-granular particles. BET and FE SEM results clearly suggest that a higher specific surface area of a composite should yield enhanced dye adsorption onto the composite and consequently improved dye photodegradation.

### 3.6. XRD Characterization

**Figure 4:**
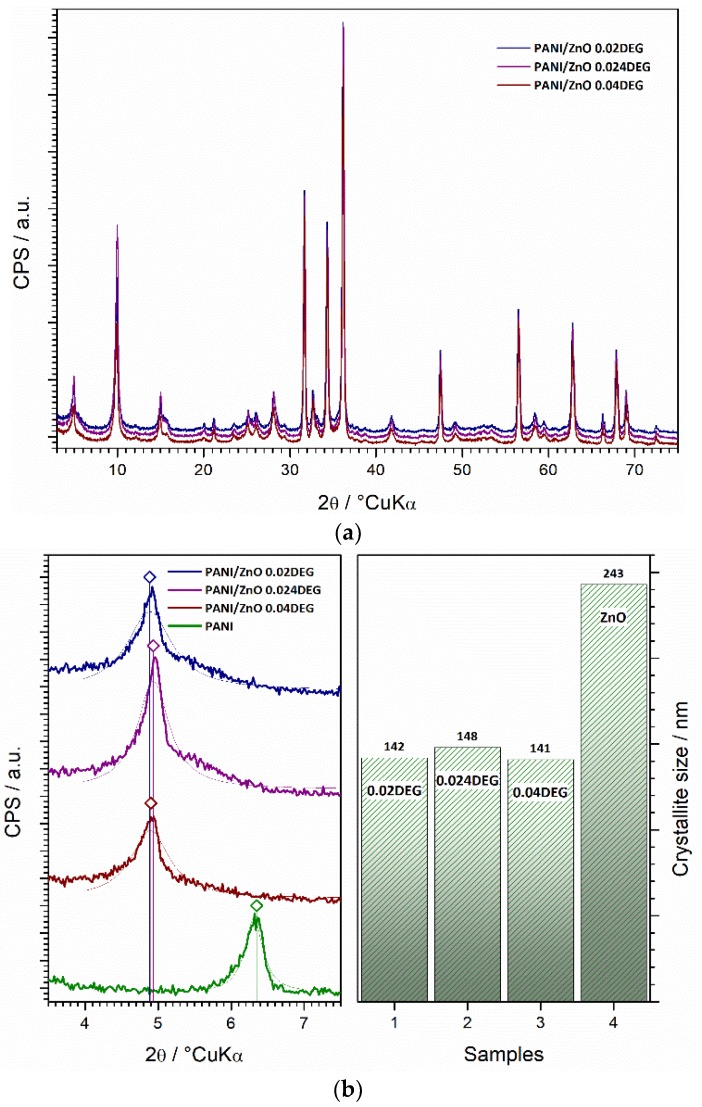

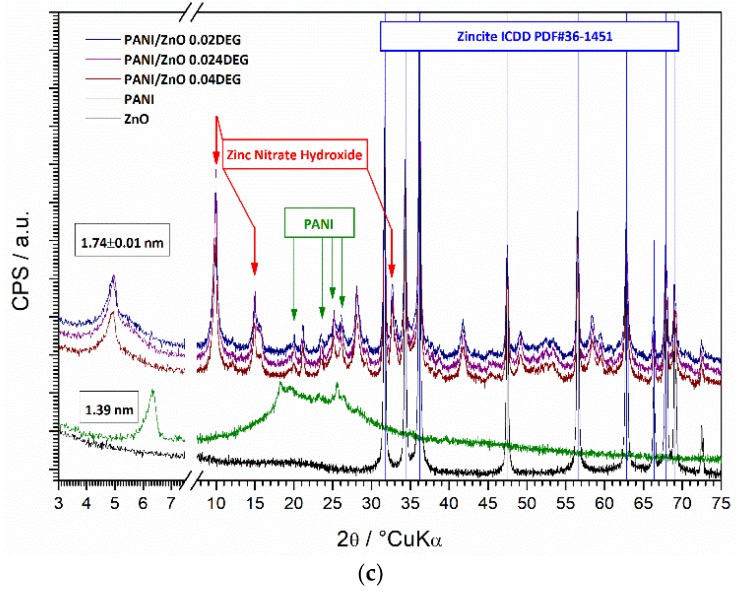
(**a**) Diffractograms of the PANI/ZnO composites, (**b**) qualitative analysis and observation of the mesostructural peak in PANI/ZnO composites, (**c**) structural parameters of the PANI/ZnO composites.

X-ray diffractograms show that the PANI/ZnO samples consist of crystalline phases, among which dominates the zincite (ZnO) pattern (ICDD PDF#36-1451) in a hexagonal P63 mc wurtzite type of structure (Figure 4). The other phase can be assigned to a conductive semi-flexible rod PANI polymer with a considerable level of structural ordering, i.e., crystallinity [47,48]. Due to the in situ synthesis conditions (i.e., usage of the strong oxidative agent (APS) and stirring), the diffractograms also show side-product and/or residual phases that resemble (zinc) nitrate-hydroxide-hydrate phase or possibly phases.

The composite constituents were also separately recorded (Figure 4a–c). ZnO powder yields a single phase diffractogram with peak shapes that suggest strongly crystalline phase with crystallite sizes (about 250 nm) that are higher compared to the ones (140–150 nm) in the composite. On the other hand, PANI powder shows a low level of structural ordering with a clearly observable mesostructural peak (6.3° 2*θ*), which corresponds to interplanar distances of 1.39 nm. However, there are only minute quantitative and broadening differences between composites and no differences in shifting. This indicates that in situ polymerization has not induced the ZnO crystal structural changes. The same cannot be said for PANI, as it clearly undergoes structural changes at a higher-order level due to interaction with ZnO.

Furthermore, the rod-like configuration of polymeric chains is obviously infiltrated by ZnO particles that extend to the interplanar distances, giving rise to a mesostructural peak at lower 2*θ* values. Interestingly, PANI/ZnO composites retain the effect characteristic for a higher level of mutual microstructural ordering, which is visible from the presence of mesostructural peaks at ≈5° 2*θ*. The composites’ peak positions correspond to interplanar distances of about 1.74 ± 0.01 nm, which is a higher value when compared to pure PANI (1.39 nm).

The retained intensity ratio of the ZnO reflection indicates that the preferred orientation is excluded. One can assume that ZnO crystallites are predominately isotropic. PANI reflections seem a bit broader and retain less of the intensity ratio, which points to smaller, less isotropic crystallites. Again, minute changes in shift and intensity of the mesostructured reflection point to the fact that initial compositional changes in composites (stoichiometry) can hardly contribute to the extent of mesostructural ordering.

Insight into lattice strain parameters may be additionally offered. We can conclude that the PANI/ZnO composite yields a synergetic effect with respect to mesostructural ordering, which may be well-utilized in sorption or ion-exchange performance in catalysis. The investigation of morphology by means of FE SEM gave additional insight. It seems that the composite poses on large micron-seized plate-like (two-dimensional, 2D) particles of PANI separated, or better to say intercalated, with relatively isotropic, partially agglomerated ZnO nanoparticles about 200 nm in size. PANI size and ZnO agglomeration varies for samples prepared with different DEG amounts; however, it seems that a lower DEG amount strongly favors a ZnO-nanoparticles-intercalated PANI-plate-like morphology, which is in concordance with the XRD results.

### 3.7. UV-Vis Spectrophotometry Characterization

**Figure 5:**
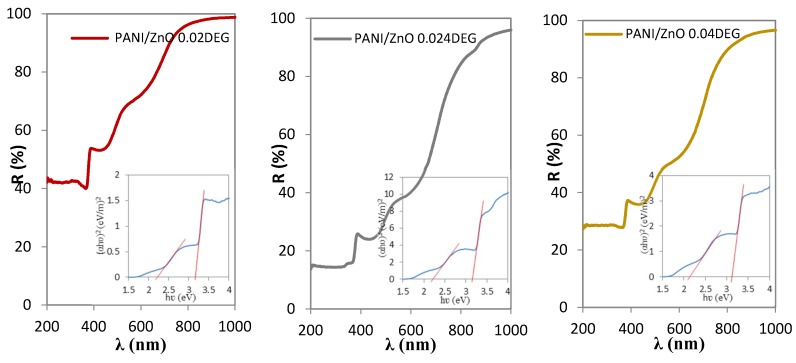
Ultraviolet (UV)-Vis reflectance spectra of the prepared PANI/ZnO composites. The insert shows the plot of (*αhυ*)^2^ versus photon (*hυ*).

The oxidation state of PANI in composites and the photocatalysts’ energy bandgap were studied by using UV-Vis spectroscopy. UV-Vis diffuse reflectance spectra (Figure 5) of PANI/ZnO composites were recorded in the wavelength range from 200 to 1000 nm. As observed, the diffuse reflectance spectra show the fundamental absorption band in the UV region and absorption bands in the visible wavelengths. The absorption band in the UV region describes the optical activity of both ZnO (<390 nm) and PANI (353 nm), whilst in the visible light range the recorded bands are the result of PANI’s optical properties [48].

Furthermore, optical ZnO and PANI bandgaps were estimated from the insert plots presented in Figure 5 [49]. The values of the bandgaps were from 2.13 to 2.22 eV for PANI at the absorbance wavelength of 423 nm and from 3.11 to 3.16 eV for the pure ZnO (Table 5). Synthesized composite photocatalysts have a lower bandgap compared to pure ZnO (3.2 eV) [50], which confirms interaction between PANI and ZnO. Thus, the in situ synthesis was successful as PANI was in its conductive form whilst dissolution of ZnO was hindered.

According to the literature [51,52,53], doped polyaniline has strong absorption for visible light as it has an optical bandgap of 2.21 eV. Table 5 shows that DEG addition resulted in a slight PANI bandgap decrease (from 2.22 to 2.13 eV), which suggests that the improved aniline solubility and better suspension stability ensured more compact interaction between PANI and ZnO. So, the absorbance of the visible light is attributed to the small bandgap and large absorption coefficient of the composites. Taking into account the efficient use of visible light in a large part of the solar spectrum, it can be assumed that PANI/ZnO composites will provide an attractive photocatalyst for degradation of a pollutant in water.

### 3.8. Photocatalytic Activity of the PANI/ZnO Composite

**Figure 6:**
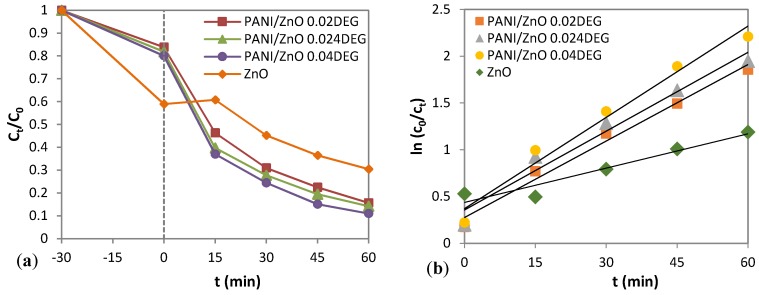
(**a**) Photodegradation of acid blue (AB25) dye under solar irradiation, (**b**) linear plots of ln (c/c_0_) versus irradiation time.

The prepared PANI/ZnO composite photocatalysts were validated by monitoring the degradation of AB25 dye in water (30 mg/L) under simulated solar irradiation using 1 g/L of catalysts. The pH of suspension during photocatalysis was adjusted to 7 due to the sensitivity of ZnO on the pH media. Figure 6a) shows AB25 dye removal from water obtained by using both the pure ZnO and different PANI/ZnO composites. Prior to exposing the dye suspension to irradiation, the adsorption process was carried out in the dark for 30 min to reach adsorption-desorption equilibrium.

During the adsorption process, PANI/ZnO composite samples removed ≈20%, whereas the pure ZnO removed ≈40%, of AB25 dye (Figure 6a). Next, after 60 min of photocatalysis, 84%, 86%, 89%, and 70% of the dye was removed by the PANI/ZnO 0.02 DEG, 0.024 DEG, 0.04 DEG, and ZnO catalysts, respectively. Furthermore, it can be concluded that the main DEG effect in this work was to prevent the ZnO’s dissolution. However, improved PANI/ZnO 0.04 DEG photodegradation properties can be attributed to a higher specific surface area (see Table 4), which was obtained as DEG addition yielded both improved aniline solubility and a more stable synthesis suspension. The removal of AB25 dye clearly confirms the higher photoactivity of PANI/ZnO (versus ZnO) catalysts under solar irradiation. It can be concluded that PANI boosted the PANI/ZnO composite’s photocatalytic properties and the same effect was observed in our previous work when using PANI/TiO_2_ [41].

**Scheme 3:**
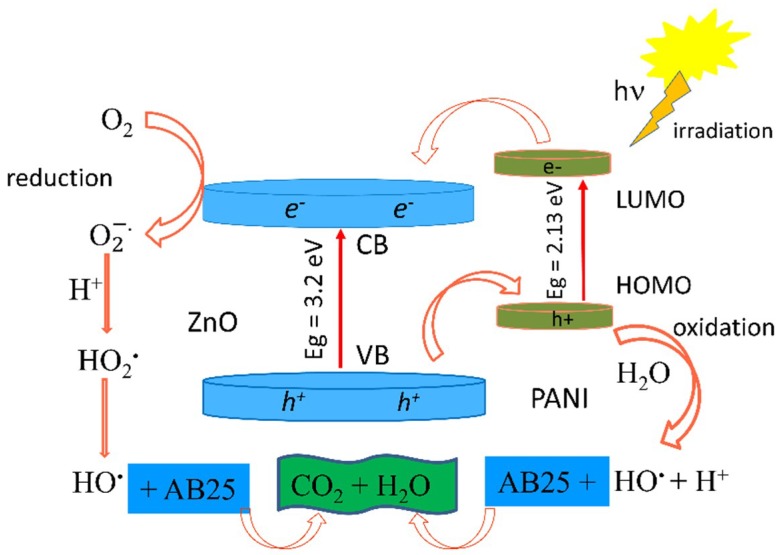
Proposed photocatalytic degradation mechanism of AB25 using PANI/ZnO composites. CB, conducting band; VB, valence band; LUMO, lowest unoccupied molecular orbital; HOMO, highest occupied molecular orbital.

The photocatalytic mechanism of dye degradation is presented in Scheme 3. The photogenerated electrons in PANI transit from the valence band (HOMO, π-orbital) to the conducting band (CB) (LUMO, p*-orbital) and they can be easily injected into the CB of ZnO because their energy levels are well-matched. Synchronously, the formation of electron–hole pairs takes place and will react with water and oxygen to produce reactive oxygen species ($O_{2}^{-∙}$, ${\mathrm{HO}}_{2}^{∙}$, ${\mathrm{OH}}^{∙}$).

The most important species for the photocatalytic process are hydroxyl radicals (${\mathrm{OH}}^{∙})$. formed by reaction between the positive holes and the hydroxyl ions. Such photogenerated holes are known to be one of the strongest oxidizing agents (E = + 2.80 eV) [54]. Thus, these holes directly decompose the organic pollutant adsorbed on the surface of ZnO and they also degrade the organic pollutant in aqueous solution indirectly by forming ${\mathrm{OH}}^{∙}$ radicals. Hence, the photocatalytic degradation can almost mineralize all organic pollutants that finally become CO_2_ and H_2_O [55,56,57].

The results in Table 6 and Figure 6b present the kinetics parameters of the photocatalytic degradation rate of AB25 dye with PANI/ZnO composite catalysts. The degradation of organic pollutant in wastewater with a semiconducting oxide usually follows the Langmuir-Hinshelwood mechanism [58].

ln(*c*_0_/*c*_t_) = *k_app_ t* (9)

where *k_app_* is the apparent rate constant, and *c*_0_ and *c*_t_ are the concentration of dye after 30 min of adsorption (in the dark) and the concentration of dye at time *t*, respectively.

It follows that the relationship between the irradiation time and degradation rate of AB25 dye under solar light is a straight line (Figure 6b). This linear correlation (see ln(*c*_0_/*c*_t_) versus *t* data) suggests a pseudo-first-order reaction for all studied composite catalysts. By comparing the obtained *k_app_* values (Table 6), the photocatalytic efficiency of studied composites can be estimated. As one can observe, the *k_app_* value increases with higher DEG addition, which indicates that DEG additions enhanced the photocatalyst’s properties by inducing variation in the composite catalyst’s structure.

The advantages of the presented approach to preparing PANI/ZnO composites are both enhanced visible light absorption (Table 6) and the ability to prevent dissolution of ZnO particles due to synthesis from neutral/slightly acidic media [19]. Enhanced PANI/ZnO interactions in composites are confirmed by the obtained lower energy gap. As PANI encapsulated ZnO particles, it additionally protected them from dissolution, i.e., PANI “captured” protons that occurred during in situ polymerization [59]. DEG improved the aniline solubility and polymerization suspension stability; thus, it enabled the formation of a spherical rather than a lamellar structure (Figure 3). The presence of a spherical structure enhanced the adsorptive capacity of the catalyst; and, consequently, the synergistic effect which is of the utmost importance for the photocatalytic efficiency. The assumption is that the presence of DEG perturbed the hydrophilic hydrophobic ratio which induced the formation of a spherical or lamellar structure [60]. It is important to stress that the degree of photodegradation of AB25 dye was significantly higher than that of the pure ZnO catalyst.

## 4. Conclusions

It was presented that it was possible to prepare PANI/ZnO composites by in situ polymerization from neutral media with the addition of DEG. FE SEM and TG analyses confirmed that ZnO was not dissolved during the preparation process. FTIR and electric conductivity results confirmed that PANI was in its conductive form.

The conducted in situ polymerization yielded both a lower bandgap of composites and a PANI/ZnO synergistic effect. The synergistic effect was also confirmed by monitoring the photodegradation of AB25 dye in wastewater.

According to FE SEM images and BET measurements, the presence of DEG during in situ PANI/ZnO synthesis yielded variations in the morphology and in a specific area of the catalyst. DEG additions increased the fraction of spherical-granular particles (versus lamellar structure), which increased the number of common spots between PANI and ZnO (i.e., it boosted interactions between constituents).

This presented approach showed significant progress in the prevention of ZnO’s dissolution during in situ PANI/ZnO photocatalyst synthesis. Thus, the same approach should be used when preparing PANI composites with metal-oxides, such as ZnO, that are sensitive to low pH.

## Figures and Tables

**Table 1:** The components used for the preparation of the polyaniline/zinc oxide (PANI/ZnO) composite photocatalysts.

Sample	PANI/ZnO 0.02 DEG	PANI/ZnO 0.024 DEG	PANI/ZnO 0.04 DEG
m(PANI):m(ZnO)%	n(ANI):n(APS) = 1:0.25		
w(ANI/ZnO)/%	15/85	15/85	15/85
m(ZnO)/g	0.800	0.800	0.800
V(ANI)/mL	0.588	0.588	0.588
m (APS)/g	0.367	0.367	0.367
V(DEG)/mL	0.386	0.466	0.777

DEG, diethylene glycol; APS, ammonium persulfate.

**Table 2:** Conductivity (κ) of PANI/ZnO composites and pure ZnO.

**Sample**	PANI/ZnO 0.020 DEG	PANI/ZnO 0.024 DEG	PANI/ZnO 0.040 DEG	ZnO
**κ/Scm^−1^**	1.862 × 10^−6^	1.935 × 10^−6^	1.691 × 10^−6^	2.955 × 10^−7^

**Table 3:** Data obtained from thermal analysis of PANI/ZnO composites: *T*_95_ and *T_max_*_1,2,3,4_, weight loss for each specific temperature region (**Δ***m*_1,2,3,4_), and the chair residue (*m*_f_).

**Samples**	***T*_95_**	***T_max_*_1_**	***T_max_*_2_**	***T_max_*_3_**	***T_max_*_4_**
	**°C**				
PANI/ZnO 0.020 DEG	254.5	72.3	268.8	364.9	541.2
PANI/ZnO 0.024 DEG	254.4	63.2	275.5	368.1	536.9
PANI/ZnO 0.040 DEG	245.4	70.3	266.8	365.6	529.5
	*m*_f_ *	**Δ*m*_1_**	**Δ*m*_2_**	**Δ*m*_3_**	**Δ*m*_4_**
	**%**				
PANI/ZnO 0.020 DEG	78.2	3.4	6.3	0.4	8.4
PANI/ZnO 0.024 DEG	77.5	3.1	6.1	0.7	8.3
PANI/ZnO 0.040 DEG	76.7	3.4	6.4	0.5	8.4

* mass left at 700 °C.

**Table 4:** Specific surface area (*S*_BET_) of PANI/ZnO composites.

Samples	*S*_BET_ (m^2^/g)
PANI/ZnO 0.02 DEG	4.4409 ± 0.0559
PANI/ZnO 0.024 DEG	5.1713 ± 0.0433
PANI/ZnO 0.04 DEG	5.6680 ± 0.0417

**Table 5:** Energy bandgap (*E_g_*) determined from ultraviolet (UV)-Vis reflectance spectra.

Samples	*E_g_* PANI (eV)	*E_g_* ZnO (eV)
PANI/ZnO 0.02 DEG	2.22	3.16
PANI/ZnO 0.024 DEG	2.16	3.16
PANI/ZnO 0.04 DEG	2.13	3.11

**Table 6:** Apparent rate constants (*k_app_*) of dye degradation and linear regression coefficients extracted from ln(*c*_0_/*c*_t_) − *t* plots of studied samples.

Samples	*R* ^2^	*k_app_* (min^−1^)
PANI/ZnO 0.02 DEG	0.9844	0.0272
PANI/ZnO 0.024 DEG	0.9662	0.0281
PANI/ZnO 0.04 DEG	0.9753	0.0325
ZnO	0.9273	0.0062
